# Identification of Transient Noise to Reduce False Detections in Screening for Atrial Fibrillation

**DOI:** 10.3389/fphys.2021.672875

**Published:** 2021-06-04

**Authors:** Hesam Halvaei, Emma Svennberg, Leif Sörnmo, Martin Stridh

**Affiliations:** ^1^Department of Biomedical Engineering, Lund University, Lund, Sweden; ^2^Department of Medicine, Karolinska Institutet, Stockholm, Sweden

**Keywords:** short-term ECG signals, transient noise, signal quality, handheld devices, mass screening, convolutional neural network

## Abstract

Screening for atrial fibrillation (AF) with a handheld device for recording the ECG is becoming increasingly popular. The poorer signal quality of such ECGs may lead to false detection of AF, often caused by transient noise. Consequently, the need for expert review in AF screening can become extensive. A convolutional neural network (CNN) is proposed for transient noise identification in AF detection. The network is trained using the events produced by a QRS detector, classified into either true beat detections or false detections. The CNN and a low-complexity AF detector are trained and tested using the StrokeStop I database, containing 30-s ECGs from mass screening for AF in the elderly population. Performance evaluation of the CNN-based quality control using a subset of the database resulted in sensitivity, specificity, and accuracy of 96.4, 96.9, and 96.9%, respectively. By inserting the CNN before the AF detector, the false AF detections were reduced by 22.5% without any loss in sensitivity. The results show that the number of recordings calling for expert review can be significantly reduced thanks to the identification of transient noise. The reduction of false AF detections is directly linked to the time and cost spent on expert review.

## 1. Introduction

Mass screening using intermittent single-lead ECGs for early detection of atrial fibrillation (AF) can help identify patients with untreated AF and thereby reduce the risk of ischemic stroke by oral anticoagulation treatment (Svennberg et al., 2015; Freedman et al., 2017; Platonov and Corino, 2018). The prevalence of AF increases with age, from 1–2% in the general population to as high as 10% in the elderly (age ≥75) (Freedman et al., 2017). Hence, screening is primarily focused on the elderly population.

Thanks to recent advances in low-cost technology for recording the ECG with a handheld device (Lau et al., 2013; Tieleman et al., 2014; Vaes et al., 2014), mass screening in the home environment has become feasible (Engdahl et al., 2013; Lau et al., 2013; Kearley et al., 2014; Svennberg et al., 2015; Kaasenbrood et al., 2016; Zink et al., 2021). Screening with handheld devices may go on for weeks, resulting in multiple intermittent ECGs, each with a duration typically ranging from 30 to 60 s. However, signals recorded with a handheld device have poorer quality than clinical signals recorded at rest, mainly due to the presence of motion artifacts and poor electrode contact.

**Figure 1:**
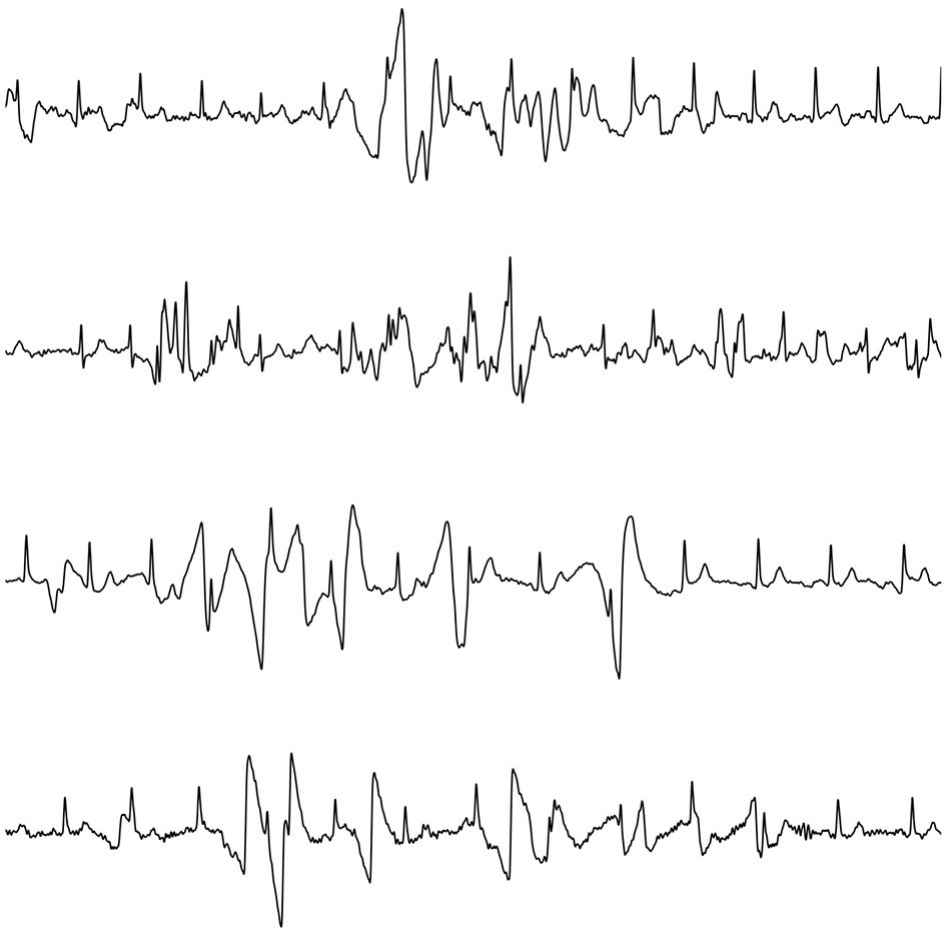
Examples of transient noise observed in ECG screening with a handheld device.

Transient noise, exemplified in Figure 1, constitutes the main source of falsely detected QRS complexes, transforming a regular rhythm into an irregular one falsely detected as AF. Since screening databases may contain up to hundreds of thousands of recordings, false AF detections cause an extensive expert review load which is time-consuming, and, therefore, very costly to deal with (Freedman et al., 2017; Svennberg et al., 2017).

The review load can be reduced by identifying and excluding noisy signals segments before further processing is performed. The identification problem has been addressed from a general ECG analysis perspective in many studies (Ghaffari et al., 2010; Clifford et al., 2012; Hayn et al., 2012; Behar et al., 2013; Orphanidou et al., 2015; Daluwatte et al., 2016; Abdelazez et al., 2017; Orphanidou and Drobnjak, 2017; Yaghmaie et al., 2018; Moeyersons et al., 2019; Huerta-Herraiz et al., 2020; Smital et al., 2020), however, only a few studies have done so in relation to AF detection (Oster and Clifford, 2015; Taji et al., 2018; Bashar et al., 2019). Then, the methods for identifying poor-quality segments have been based on comparing the output of two different QRS detectors (one being more sensitive to noise than the other) (Oster and Clifford, 2015), deep belief networks (Taji et al., 2018), and time–frequency analysis combined with sub-band decomposition of the ECG signal (Bashar et al., 2019); the latter two studies did not rely on QRS detection. In these three studies, the ability to identify poor-quality segments was evaluated on long-term recordings, using either the Physionet Long-Term AF Database (Oster and Clifford, 2015), a subset of the MIT–BIH AF Database (Taji et al., 2018), or a subset of the MIMIC III database (Bashar et al., 2019). By adding noise to the ECG recordings, AF detection performance could be presented as a function of the signal-to-noise ratio in Oster and Clifford (2015) and Taji et al. (2018). Noise typical of signals obtained from screening in the home environment was not considered in any of these three studies.

The present study proposes and evaluates a novel method for deep learning-based quality control in AF detection, with the ultimate goal to reduce the number of recordings requiring expert review. The quality control, inserted between the QRS detector and the AF detector, is accomplished by a convolutional neural network (CNN), trained using good- and poor-quality recordings. Transient noise is identified by the CNN on an event-to-event basis, meaning that the events produced by the QRS detector are classified as either true beat detections, i.e., heartbeats, or false detections, i.e., noise. The proposed method is developed and tested using different subsets of the StrokeStop I database (Svennberg et al., 2015). To the best of our knowledge, this study is the first to establish the degree of improvement in AF detection performance when using CNN-based quality control^1^.

The remainder of this paper is organized as follows: section 2 describes the database and types of annotation. Section 3 describes the proposed method for quality control and the AF detector. The results are presented in section 4, then subjected to discussion in section 5.

## 2. Database and Annotations

The StrokeStop I database is divided into two parts (denoted SSI-A and SSI-B) depending on whether or not expert annotation is provided:

- SSI-A contains 81,063 lead-I ECG recordings from 3,209 75- or 76-year old subjects. Expert annotation is provided using the two categories AF and non-AF, assigned to 259 and 80,804 recordings, respectively.
- SSI-B contains the remaining part of the StrokeStop I database with 114,138 recordings from 3,964 75- or 76-year old subjects. Since no expert annotation is provided, this part was machine annotated, see below.

The ECGs were recorded using Zenicor handheld ECG devices (Zenicor Medical System AB, Sweden) and transmitted to a center for offline analysis. The recording duration is 30 s. For each subject, an average of 26 ECGs were recorded over a period of 2 weeks. Recordings with at least 10 s of AF were, as a whole, annotated as AF (Svennberg et al., 2015).

The database was approved by the Ethical Review Board of Karolinska Institute (211/1363-31/3) after informed consent to all subjects.

Since no expert annotation was provided for SSI-B, a commercial CE-approved software for ECG analysis (Cardiolund AB, Lund, Sweden) was used to machine annotate SSI-B. The machine annotation resulted in the following four categories: *normal rhythm, irregular rhythm, other rhythm* (i.e., bigeminy, trigeminy, multiple ventricular/supraventricular ectopic beats, fast/slow sequence, pause/AV blocks), and *noise*, having the composition presented in Table 1.

**Table 1:** SSI-A and SSI-B composition after machine annotation.

	**SSI-A (%)**	**SSI-B (%)**
Normal rhythm	86.9	84.2
Irregular rhythm	6.9	7.7
Other rhythm	4.0	4.4
Noise	2.2	3.7

Since the aim of the present study is to reduce the number of false AF detections, the performance of the proposed approach is evaluated on recordings which are likely to cause false AF detections and therefore requiring expert review. Typically, such recordings are *machine annotated* by the category *irregular rhythm* containing the following entities: AF, irregular rhythms not part of the category *other rhythm*, and false irregular rhythms caused by transient noise. Since SSI-A was expert annotated, therefore lacking the category *irregular rhythm*, SSI-A was also machine annotated to facilitate the creation of the dataset used for evaluating AF detection performance (see section 3.3). Note that the above notion “irregular rhythm not part of the category *other rhythm*” refers to recordings mainly containing irregular rhythms, such as short episodes of supraventricular tachyarrhythmias and runs of ectopic beats.

## 3. Methods

**Figure 2:**
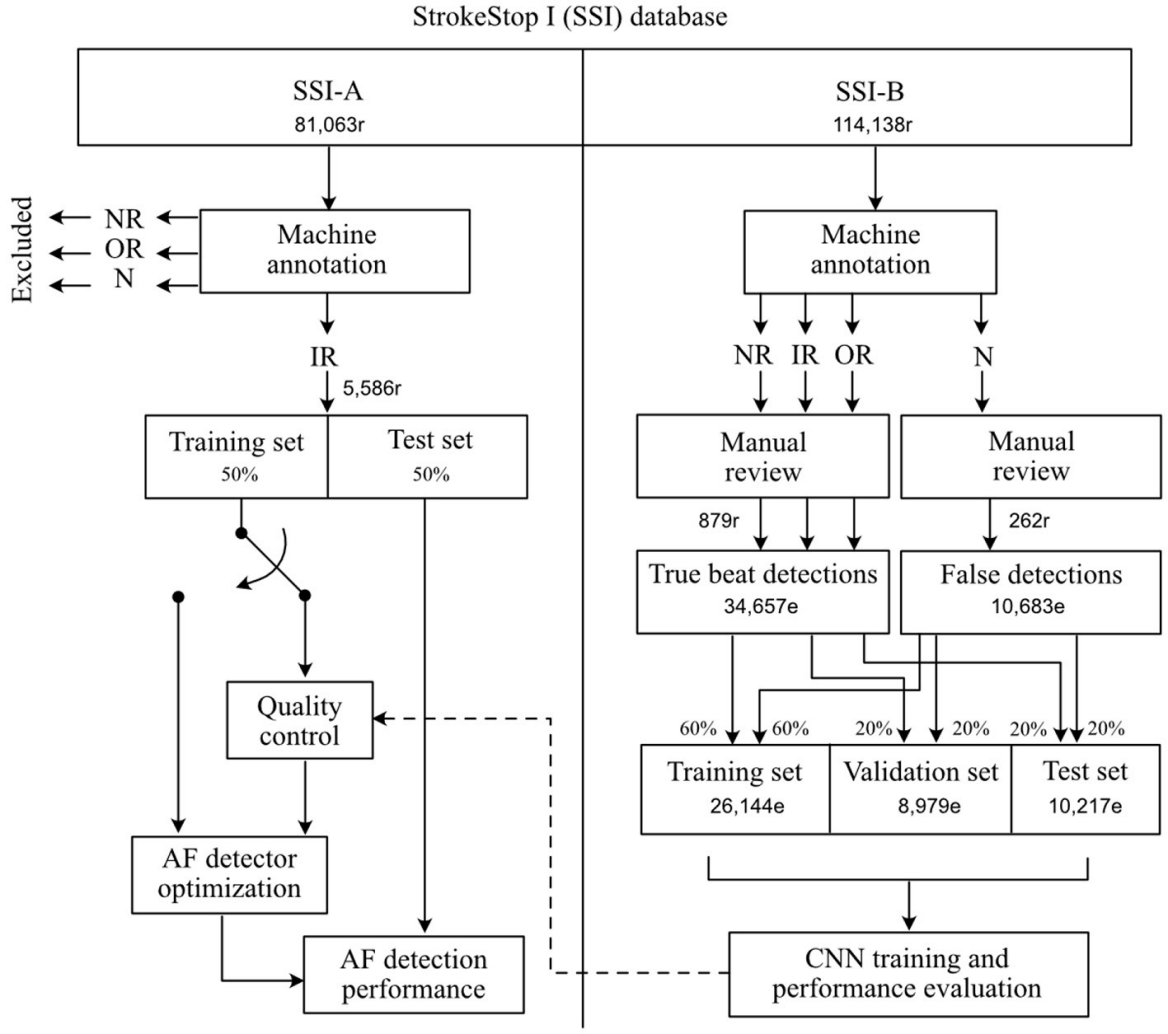
Creation of datasets for CNN-based quality control and AF detection training and performance evaluation. The machine annotated categories *normal rhythm, irregular rhythm, other rhythm*, and *noise* are abbreviated to NR, IR, OR, and N, respectively. The number of recordings are indicated by an appended “r” and the number of events are indicated by an appended “e”.

The proposed approach to quality control involves the following steps: (1) Creation of training, validation, and test datasets for CNN-based quality control, (2) Training of the CNN, (3) Creation of training and test datasets for AF detection, and (4) Optimization of the AF detector parameters. A block diagram, showing creation of the datasets used for training and performance evaluation of the CNN and AF detector is presented in Figure 2.

### 3.1. CNN Training, Validation, and Test Datasets

**Figure 3:**
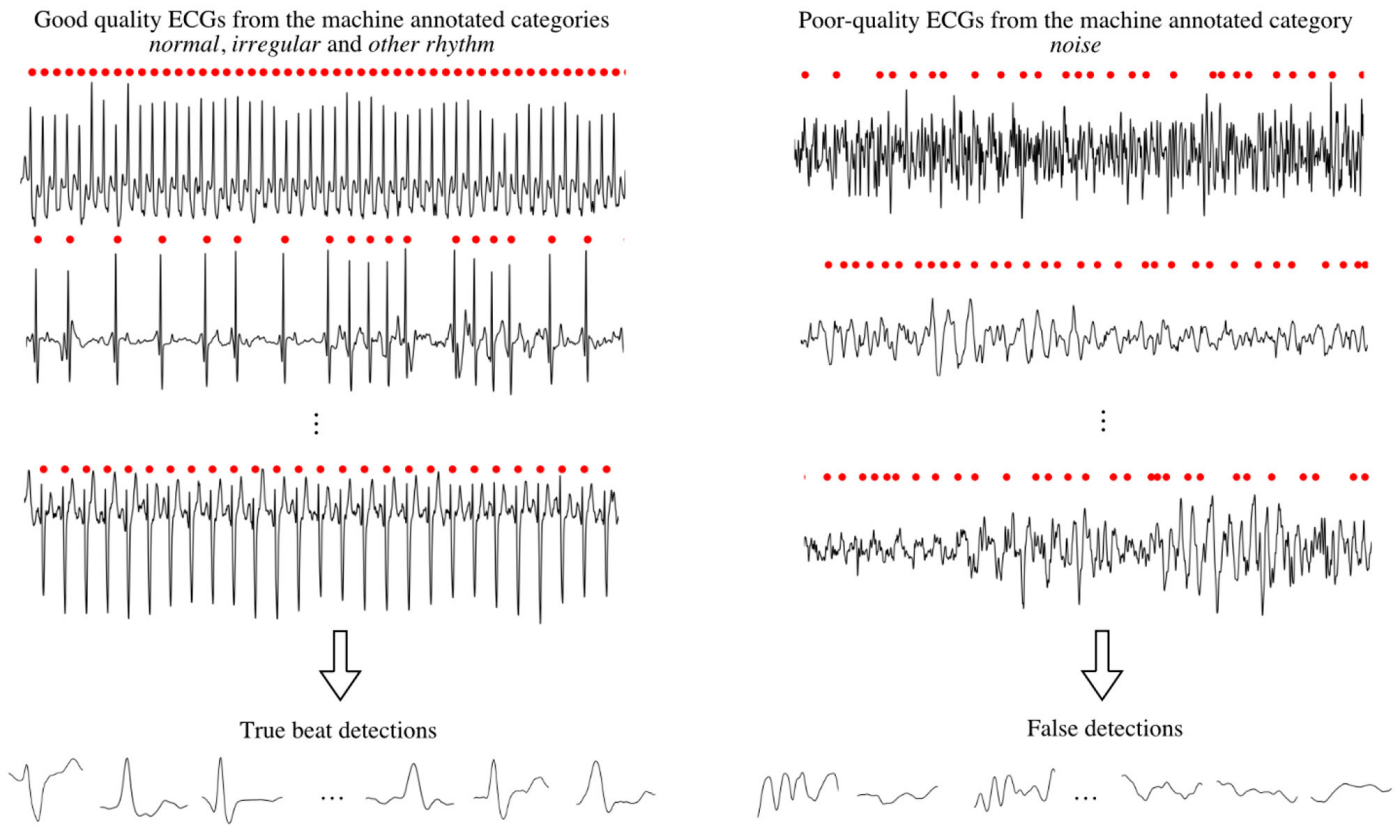
Compiling the events, i.e., true beat detections and false detections, for CNN training, validation, and testing.

The task of the CNN is to perform quality control on an event-to-event basis. Using SSI-B, a large number of *true beat detections* and *false detections* were compiled, from good-quality and poor-quality ECGs, respectively (see Figure 3). Each event is defined by a 400-ms segment (sampling rate of 1,000 Hz), 150 ms before and 250 ms after the occurrence time produced by the built-in QRS detector of the commercial software. The events were scaled to the range in [0, 1] using min-max normalization.

A total of 34,657 true beat detections were selected from 879 recordings in the machine-annotated categories *normal rhythm, irregular rhythm*, and *other rhythm*. The recordings were manually reviewed to ensure that no false detections were included.

A total of 10,683 false detections were selected from 262 recordings in the machine annotated category *noise*. Again, the selected recordings were manually reviewed but now to ensure that no true beat detections were included.

Together, the recordings of these two groups make up for 1% of the total number of recordings in SSI-B.

The training, validation, and test sets were created by 60, 20, and 20%, respectively, of the true beat detections and false detections. It should be noted that no patient appeared in more than one of the training, validation, and test sets.

### 3.2. CNN Training and Architecture

Several CNN architectures were tested and different experiments were carried out to determine a satisfactory combination of the number of convolutional layers, fully connected layers, and pooling layers. In addition, different kernel size, stride size, dropout rate, batch size, and learning rate were examined. The search space for determining the best CNN architecture and fine-tuning is given in Table 2.

**Table 2:** CNN architecture and fine-tuning search space.

Number of convolutional layers	[1 2 3 4]
Number of fully-connected layers	[1 2 3 4]
Number of kernels	[8 16 32 64]
Kernel size	[5 10 15]
Stride size	[1 2 3]
Number of neurons	[10 15 20 25 30 35 40 45 50]
Dropout rate	[0.2 0.25 0.3 0.35 0.4 0.45 0.5]
Learning rate	[0.01 0.03 0.001 0.003]
Batch size	[128 256 512 1,024]

The structure of the selected network and the number of parameters are given in Table 3. Three consecutive 1-D convolutional layers with the number of filters of 16, 32, and 64, respectively, where the last two followed by an average pooling layer, are used to extract and summarize the most pertinent feature maps of the 400-ms signal segment. The kernel size in convolutional and average pooling layers is set to 10 and 5, respectively, and the stride size in both is 2. The convolutional and pooling layers are followed by three fully-connected layers with the number of neurons set to 40, 40, and 1, respectively.

**Table 3:** Definition of layers and parameters of the proposed CNN.

**Layer**	**Type of layer**	**Number of parameters**	**Number of filters/Neurons**	**Kernel size/Stride**	**Output shape**
0	Input layer	–	–	–	400 × 1
1	1D-Conv	176	16	10 / 2	196 × 16
2	1D-Conv	5,152	32	10 / 2	94 × 32
3	Average pooling	–	–	5 / 2	45 × 32
4	1D-Conv	20,544	64	10 / 2	18 × 64
5	Average pooling	–	–	5 / 2	7 × 64
6	Fully connected	17,960	40	–	1 × 40
7	Dropout (0.5)	–	–	–	1 × 40
8	Fully connected	1640	10	–	1 × 40
9	Dropout (0.25)	–	–	–	1 × 40
10	Fully connected	41	1	–	1 × 1

The number of epochs was set to 200 and the CNN was trained with a batch size of 256, using the Adam optimizer with a learning rate of 0.001.

To account for data imbalance, the weighted binary cross entropy is used as loss function, defined by:

(1) $$\begin{array}{l} L=-\frac{1}{M}\overset{M}{\underset{i=1}{\sum }}\left[w_{1}y_{i}\log\left({ŷ}_{i}\right)+w_{0}\left(1-y_{i}\right)\log\left(1-{ŷ}_{i}\right)\right], \end{array}$$

where *M* is the total number of training data, *y*_*i*_ is the label of the *i*-th training sample, and ŷ_*i*_ is its prediction. The weights *w*_0_ and *w*_1_ are associated with the numbers of true beat detections *M*_0_ and false detections *M*_1_, respectively, defined by

(2) $$\begin{array}{l} w_{j}=\frac{1}{M_{j}}\frac{M}{2},\;j=0,1. \end{array}$$

In order to avoid overfitting, two dropout layers with the rate of 0.5 and 0.25 are inserted between the first two convolutional layers. L2 regularization with penalty weight of 0.01 is applied to the first convolutional layer. In addition, at the end of each epoch, *L* is computed on the CNN validation set to stop training in case that loss increases.

The *Relu* activation function is used for the convolutional and fully-connected layers, except for the final fully-connected layer which uses a sigmoid activation function. The output sigmoid layer provides a probability, meaning that an event is identified as a false detection when its probability is higher than a certain threshold. This threshold is set to the value which maximizes the *F*_1_-score (defined in section 3.5) on the CNN validation set.

### 3.3. AF Detection Training and Test Datasets

Recordings in SSI-A, machine annotated as *irregular rhythm*, were used to train and test the AF detector. The category *irregular rhythm* of SSI-A contains 5,586 recordings from 1,548 subjects, of which 237 recordings from 77 subjects were expert annotated as AF (see Table 4).

**Table 4:** Composition of the category *irregular rhythm* in SSI-A resulting from expert annotation.

	**AF**	**Non-AF**
# of subjects	77	1,471
# of recordings	237	5,349

The category *irregular rhythm* was divided into training and test sets, where recordings from 50% of the subjects were assigned to the training set and the remaining to the test set. Given that the number of AF patients is much smaller than the number of non-AF subjects, the AF patients were divided equally between the training and the test sets. To reduce the performance bias resulting from a single data split, the evaluation was repeated 10 times using random splits.

### 3.4. AF Detection Optimization

In the present study, a variation on the low-complexity AF detector described in Petrėnas et al. (2015) is used. The detector explores the fact that AF episodes are associated with irregular RR intervals. quantified by

(3) $$\begin{array}{l} \Lambda_{i}=\frac{1}{\left(N-1\right)\left(N-2\right)}\overset{N-2}{\underset{j=0}{\sum }}\overset{N-1}{\underset{k=j+1}{\sum }}H\left(\gamma_{i}-|r_{i}\left(j\right)-r_{i}\left(k\right)|\right), \end{array}$$

where *r*_*i*_(*n*), *n* = 0, 1, …, *N*−1, denotes the RR intervals within a sliding window whose onset is positioned at the *i*-th RR interval, *H*(·) is the Heaviside step function, and *N* is the length of the sliding window. The threshold γ_*i*_ is defined by

(4) $$\begin{array}{l} \gamma_{i}=\alpha \cdot \text{median}\left[r_{i}\left(0\right),r_{i}\left(1\right),\ldots ,r_{i}\left(N-1\right)\right], \end{array}$$

where α is a constant. Whenever Λ_*i*_ exceeds the threshold η, the RR intervals in the sliding window are considered irregular:

(5) $$\begin{array}{l} O_{i}=\{\begin{array}{cc} 1 & \Lambda_{i}\geq \eta ,\\ 0 & \Lambda_{i}\leq \eta . \end{array} \end{array}$$

Finally, AF is detected whenever

(6) $$\begin{array}{l} \frac{1}{I}\overset{I}{\underset{i=1}{\sum }}O_{i}\geq \eta_{d}, \end{array}$$

where *I* is the number of sliding windows accommodated in a 30-s recording. The threshold η_*d*_ is set to 1/3 as recordings with AF episodes as short as 10 s are annotated as AF.

The parameters *N*, η, and α are optimized with and without quality control. The parameter search space is defined by 4 ≤ *N* ≤ 8, 0.03 ≤ α ≤ 0.12, and 0.05 ≤ η ≤ 0.95. Subject to the constraint that sensitivity ≥99%, the parameter values yielding the lowest false positive rate are determined; these two metrics are defined in section 3.5.

The above description builds on the assumption that the entire series of RR intervals is used for AF detection. However, with quality control, the false detections identified by the CNN need to be handled before AF detection. This is done by omitting all sliding windows containing false detections, except when a false detection occurs between normally spaced true beat detections, deviating <15% from the median RR interval; then, the false detection is omitted. It should be noted that with quality control *I* is given by the number of windows qualifying for detection.

### 3.5. Performance Evaluation

Performance is evaluated for the following three situations: CNN-based quality control, AF detection on a recording basis, and AF detection on a patient basis.

The metrics sensitivity (Se), specificity (Sp), accuracy (Acc), false positive rate (FPR), and positive predictive value (PPV) are used, defined by

(7) $$\begin{array}{l} \text{Se}=\frac{N_{TP}}{N_{TP}+N_{FN}}, \end{array}$$

(8) $$\begin{array}{l} \text{Sp}=\frac{N_{TN}}{N_{TN}+N_{FP}}, \end{array}$$

(9) $$\begin{array}{l} \text{Acc}=\frac{N_{TP}+N_{TN}}{N_{TP}+N_{FP}+N_{TN}+N_{FN}}, \end{array}$$

(10) $$\begin{array}{l} \text{FPR}=1-\text{Sp}=\frac{N_{FP}}{N_{TN}+N_{FP}}, \end{array}$$

(11) $$\begin{array}{l} \text{PPV}=\frac{N_{TP}}{N_{TP}+N_{FP}}, \end{array}$$

(12) $$\begin{array}{l} F_{1}=\frac{2\times \text{Se}\times \text{PPV}}{\text{Se}+\text{PPV}}, \end{array}$$

respectively. The interpretation of *N*_*TP*_, *N*_*FP*_, *N*_*TN*_, and *N*_*FN*_ depends on the situation in which performance is evaluated, see below.

#### 3.5.1. CNN-Based Quality Control Performance

In this case, *N*_*TP*_ is the number of false detections manually annotated as false detections, *N*_*TN*_ is the number of true beat detections manually annotated as true beat detections, *N*_*FP*_ is the number of false detections manually annotated as true beat detections, and *N*_*FN*_ is the number of true beat detections manually annotated as false detections. Sensitivity, specificity, and accuracy and *F*_1_-score are computed in this case.

#### 3.5.2. AF Detector Performance on a Recording Basis

In this case, *N*_*TP*_ is the number of recordings detected as AF and expert annotated as AF, *N*_*TN*_ is the number of recordings detected as non-AF and expert annotated as non-AF, *N*_*FP*_ is the number of recordings detected as AF and expert annotated as non-AF, and *N*_*FN*_ is the number of recordings detected as non-AF and expert annotated as AF. Sensitivity, false positive rate, and positive predictive value are computed.

#### 3.5.3. AF Detector Performance on a Patient Basis

In this case, *N*_*TP*_ is the number of detected AF patients expert annotated as AF, and *N*_*FN*_ is the number of patients detected as non-AF expert annotated as AF. Sensitivity is only computed in this case as the goal is to determine whether all AF patients are detected.

## 4. Results

### 4.1. CNN-Based Quality Control Performance

The performance of the trained CNN is evaluated on the test set described in section 3.1, containing true beat detections and false detections. Using the threshold obtained by maximizing the *F*_1_-score on the CNN validation set, i.e., 0.75, the following figures resulted: Se = 96.4%, Sp = 96.9%, Acc = 96.9%, and *F*_1_-score = 92.5% (see Table 5).

**Table 5:** CNN performance on the test set.

	**Sensitivity**	**Specificity**	**Accuracy**	***F*_1_-score**
CNN test set	96.4%	96.9%	96.9%	92.5%

**Figure 4:**
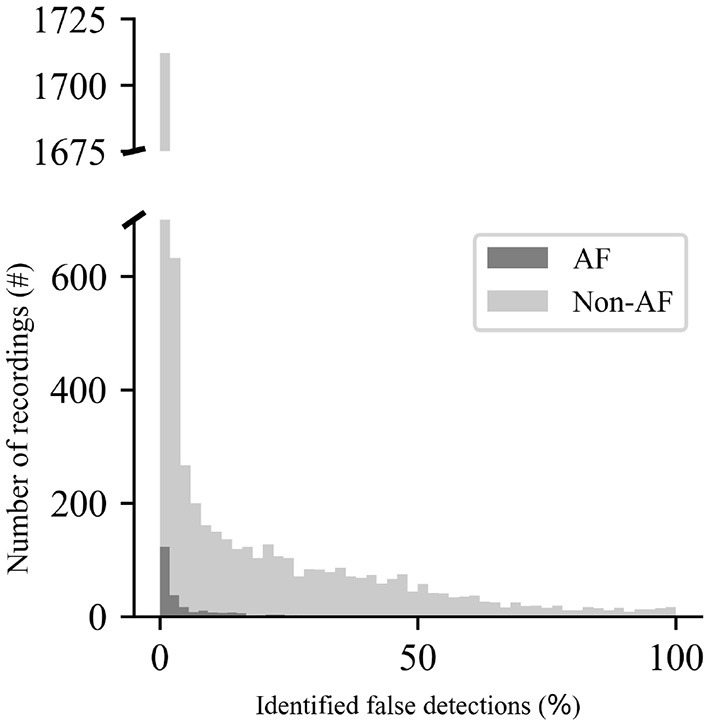
Percentage of the total number of detections belonging to the recordings in the category *irregular rhythm* identified as false detections.

The effect of applying quality control to the SSI-A recordings machine annotated as *irregular rhythm* is shown in Figure 4, presented as the percentage of the total number of events identified as false detections. Out of the 5,586 recordings annotated as *irregular rhythm*, 2,693 have at least 5% of all detections identified as false, whereas 2,893 recordings have <5%.

**Figure 5:**
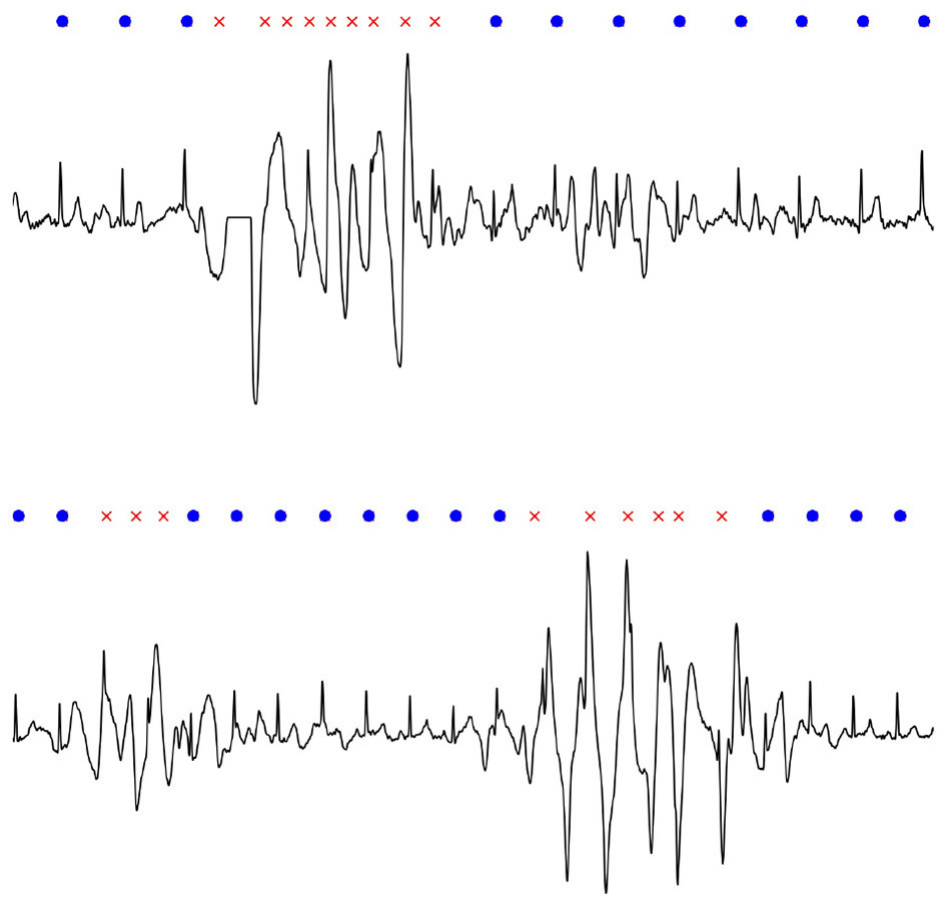
Without quality control, these two recordings are falsely detected as AF due to the rhythm irregularity caused by transient noise. By identifying and excluding transients (red crosses) before AF detection, the two recordings are correctly detected as non-AF; true beat detections are indicated with blue dots.

The performance is illustrated by two examples in Figure 5, where the many false detections are correctly excluded, but none of the true beat detections. Thanks to quality control, the AF detector correctly identifies a non-AF rhythm instead of AF, which otherwise would have been the case.

### 4.2. AF Detector Performance on a Recording Basis

Using the training sets described in section 3.3, the optimal values of *N*, α, and η are found to be 8, 0.07 and 0.55, respectively, without quality control. Not surprisingly, the optimal *N* is lowered from 8 to 4 when quality control is introduced, whereas α and η are found to be 0.04 and 0.65, respectively. Using the optimal values, the performance is determined on the 10 randomly split test sets with and without quality control (see Table 6). With quality control, a considerable improvement in FPR results, decreasing from 87.5 ± 0.7 to 65.0 ± 1.3%, without any loss in sensitivity; the PPV increases from 4.6 to 6.2%. Without quality control, this result implies that at least 22 recordings are needed for review to find one AF recording (≈100/4.6). With quality control, the corresponding number decreases to 16 (≈100/6.2).

**Table 6:** AF detection performance with and without quality control using optimal parameter values.

	**Se**	**FPR**	**PPV**
**Without quality control**			
*N* = 8, α = 0.07, η = 0.55	99.0 ± 0.6%	87.5 ± 0.7%	4.6 ± 0.4%
**With quality control**			
*N* = 4, α = 0.04, η = 0.65	99.0 ± 0.6%	65.0 ± 1.4%	6.2 ± 0.5%

The confusion matrix of a randomly sampled test set is presented in Table 7. Without quality control, the sum of *N*_*TP*_ = 106 and *N*_*FP*_ = 2,405 means that 2,511 recordings require expert review. With quality control, this number drops to 1,913 recordings. Thus, 598 fewer recordings require expert review when quality control is applied.

**Table 7:** Confusion matrix for AF detection with and without quality control.

		**AF detection outcome**			
		**Without quality control**		**With quality control**	
		**AF**	**Non-AF**	**AF**	**Non-AF**
**Expert annotation**	**AF**	106	1	106	1
	**Non-AF**	2,405	318	1,807	916

### 4.3. AF Detector Performance on a Patient Basis

Since multiple recordings are available for each subject, the 99.0 ± 0.6% sensitivity obtained for both without and with quality control (see Table 6), shows that no AF patient is missed. Thus, 100% sensitivity is achieved when evaluating performance on a patient basis.

## 5. Discussion

AF screening in the elderly population requires expert review of a huge number of recordings (Svennberg et al., 2015). The presence of transient noise in screening ECGs causes many false detections which, in turn, result in false detections of irregular rhythms. In the present study, the problem of identifying and excluding transient noise before performing AF detection is investigated. The results show that a considerable number of false AF detections can be avoided using CNN-based quality control.

### 5.1. CNN Design and Training

CNNs have found their way into various ECG applications, including arrhythmia detection (Rubin et al., 2018; Yıldırım et al., 2018; Hannun et al., 2019; Niu et al., 2020), AF detection (Andersen et al., 2019; Dang et al., 2019; Fujita and Cimr, 2019), heartbeat classification (Kiranyaz et al., 2016), QRS detection (Silva et al., 2020), and signal quality assessment (Huerta-Herraiz et al., 2020). Concerning the approach taken to signal quality assessment in Huerta-Herraiz et al. (2020), consecutive 5-s ECG segments were inputted to the CNN which assigned a label (high- or low-quality) to each segment; similar segment-based approaches were also investigated in Clifford et al. (2012) and Behar et al. (2013), but then based on traditional machine learning. In the present study, a CNN interleaved with an AF detector is proposed for quality control, differing from Clifford et al. (2012), Behar et al. (2013), and Huerta-Herraiz et al. (2020) insofar as the CNN operates on an event-to-event basis.

One reason for pursuing an event-based approach is due to the fact that atrial activity cannot be reliably analyzed in lead I, commonly recorded with a handheld device, and, therefore, rhythm-based AF detection appears as a natural choice. Another reason is that the performance of a rhythm-based AF detector depends heavily on the performance of the QRS detector (Sörnmo et al., 2018; Butkuvienė et al., 2021). An event is defined by a 400-ms window centered around the occurrence time of a QRS complex. While QRS duration and morphology can vary from subject to subject, a 400-ms window ensures that the entire heartbeat is contained in the window. The effect of inserting a CNN after QRS detection for suppression of false detections was investigated in Silva et al. (2020), also operating on an event-to-event basis but using an 800-ms window. In that study, however, only recordings without arrhythmia were analyzed, whereas, in the present study, the vast majority of recordings contain arrhythmias, making an 800-ms window unsuitable as it may contain multiple events.

The CNN was trained using true beat detections selected from good-quality recordings and false detections from poor-quality recordings. Given the huge size of SSI-B and the lack of expert annotation, a manual search for noisy signals was deemed unfeasible. Instead, machine annotation was employed to identify false detections in poor-quality recordings well-suited for CNN training. An alternative approach would have been to insert false detections by simulating transient noise to create a large, balanced dataset. However, the proposed approach for collecting false detections resulted in more than 10,000 false detections (and about 35,000 true beat detections) which was deemed sufficient. This size of dataset is similar to the one used for training, validation, and testing of a CNN for ECG-based detection of myocardial infarction (Acharya et al., 2017). In addition, a weighted loss function was used to account for data imbalance.

### 5.2. AF Detection in Mass Screening

The low-complexity AF detector is well-suited for handling short recordings obtained by a handheld device, particularly in those cases when the 30-s duration decreases due to the exclusion of false detections. The detector, offering good performance on the MIT–BIH AF Database (Petrėnas et al., 2015; Sörnmo et al., 2018), makes use of a short sliding window whose length is set to either 4 or 8 RR intervals depending on whether or not quality control is used. This length stands in contrast to the fact that most well-performing AF detectors require a much longer window, ranging from 32 to 128, with longer windows yielding better performance (Dash et al., 2009; Huang et al., 2010; Lian et al., 2011). The decisions made by the low-complexity detector can easily be traced and interpreted, while the internal rules of the CNN-based quality control distinguishing good-quality ECGs from noise have yet to be established. However, knowledge on what characterizes transient noise is of much less interest than that which characterizes AF.

Since the primary goal of mass screening is to detect all AF patients, a sensitivity very close to 100% is essential. While the achieved sensitivity on the 10 randomly split test sets was 99.0 ± 0.6% (cf. Table 6), no AF patient was missed thanks to the availability of multiple recordings.

The main result of the present study is that the FPR is reduced by as much as 22.5%, i.e., from 87.5 to 65.0%. This reduction has particular clinical significance as it is achieved on a dataset which required expert review to confirm the presence of AF.

For comparison, AF detection performance was evaluated on the PhysioNet/CinC Challenge AF database (Clifford et al., 2017) using recordings machine annotated as *irregular rhythm* (and thus requiring expert review); recordings with inverted measurements were omitted as the CNN was not trained on such recordings. Without quality control, the sensitivity was 98.7% and the FPR 83.0%. With quality control, the sensitivity was slightly reduced to 98.0% while the FPR dropped to 70.1%, i.e., a reduction of 12.9% to be compared with the 22.5% obtained on our dataset.

The lower false alarm reduction obtained on the Challenge database may be explained by the following two reasons: the SSI database is a large mass-screening database with 195,000 recordings from more than 7,000 75- and 76-year old subjects, while the Challenge database is compiled of recordings from an unknown population and preselected for the challenge. Hence, the Challenge database is not directly comparable regarding the presence of transient noise. Another reason is that the SSI database required at least 10 s of AF to be expert annotated as AF. Since no such criterion has been declared for the Challenge database, applying a detector trained on the SSI database is likely not optimal.

### 5.3. Comparison to Studies on ECG Quality Assessment

As mentioned in section 1 many methods assessing signal quality have been proposed over the years. Performance has been quantified by comparing how well the assessment agrees with annotated poor-quality segments of one or several databases. In one of those studies, the ECGs obtained from the intensive care unit were analyzed and signal quality assessed in 10-s segments to determine the extent with which poor-quality signals cause arrhythmia alarms (Behar et al., 2013). The authors came to the important conclusion that quality assessment should be rhythm-specific. However, when evaluating performance, segments with transient noise were omitted before computing the results due to probable label disagreement between the annotators.

The earlier mentioned CNN-based approach for detecting poor-quality segments (Huerta-Herraiz et al., 2020) analyzed three different databases with ECGs from wearable devices. The CNN was better in discriminating high-quality from low-quality ECGs than the method in Clifford et al. (2012). The percentage of segments labeled as AF when classified as high quality were presented in Huerta-Herraiz et al. (2020), but no information on AF detection performance.

The above-mentioned approaches to signal quality assessment analyze and classify entire 5- or 10-segment, whereas the present approach operates on an event-to-event basis. While quality assessment on an event-to-event basis has been used before, e.g., to compute a set of heuristic, event-related parameters reflecting signal quality (Hayn et al., 2012) or a dynamic signal quality index (Yaghmaie et al., 2018), its significance in AF detection has not been the subject of investigation.

Interestingly, few studies have been published investigating the influence of poor signal quality on the subsequent rhythm analysis. With the aim to reduce the number of false AF detections, a time–frequency technique was employed to detect various types of artifacts (Bashar et al., 2019). The 94% reduction in false AF detections, reported in that study, is impressive when compared to the much more modest 22.5% reduction in the present study. However, a comparison is not meaningful since the present results are obtained on a subset of recordings which required expert review, whereas no such criterion was applied in Bashar et al. (2019) when creating the MIMIC III subset.

### 5.4. Limitations

A limitation is that the CNN is trained on detections from lead I, and, therefore, needs to be re-trained if another lead is to be processed. Another limitation is that the performance of the proposed method for quality control is not compared to that of any other method. This is due to the lack of studies investigating the effect of transient noise identification on AF detection performance.

## 6. Conclusions

This paper presents a CNN-based approach to identifying and excluding transient noise, being a major cause of false alarms and extensive expert review in mass screening. The reduction of false AF detections by 22.5% in the elderly population was achieved on a subset in which AF is difficult to distinguish from non-AF, and, therefore, typically require expert review. The reduced number of false AF detections translates to lower review burden and, accordingly, lower cost.

## Data Availability Statement

The data analyzed in this study is subject to the following licenses/restrictions: the approval does not permit sharing. Requests to access these datasets should be directed to hesam.halvaei@bme.lth.se.

## Ethics Statement

The studies involving human participants were reviewed and approved by Ethical Review Board of Karolinska Institute (211/1363-31/3). The patients/participants provided their written informed consent to participate in this study.

## Author Contributions

HH contributed to the concept, computer code and analysis, and manuscript draft. ES contributed to the data collection. LS and MS contributed to the concept and editorial supervision. All authors contributed to the article and approved the submitted version.

## Conflict of Interest

The authors declare that the research was conducted in the absence of any commercial or financial relationships that could be construed as a potential conflict of interest.

## Footnotes

^1^An early version of the present method and preliminary results obtained on another database were presented in Halvaei et al. (2020).
